# A CROSS-SECTIONAL ASSESSMENT OF PHARMACISTS’ KNOWLEDGE, ATTITUDE AND PRACTICE OF PREVENTION OF MOTHER-TO-CHILD TRANSMISSION OF HIV IN TWO NIGERIAN TEACHING HOSPITALS

**DOI:** 10.21010/Ajid.v16i2S.3

**Published:** 2022-08-17

**Authors:** Isah Abdulmuminu, Abubakar Mustapha Muhammed, Igboeli Nneka Uchenna, Ibezim Isaac Chijioke, Ibenekwu Chisom Sandra

**Affiliations:** 1 Department of Clinical Pharmacy and Pharmacy Management, University of Nigeria, Nsukka, Enugu State, Nigeria; 2 Directorate of Profession-Allied Medicine, Medical Services Branch, Nigerian Air Force

**Keywords:** Attitude, HIV, Knowledge, Mother-to-child, Pharmacists, Practice, Prevention, Transmission

## Abstract

**Background:**

The pharmacological component of prevention of mother-to-child transmission (PMTCT) services involves the provision of antiretroviral agents (ARVs) to the mothers and/or their babies at any stage of pregnancy. This study assessed the knowledge, attitude and practice (KAP) of Pharmacists about PMTCT.

**Materials and Methods:**

A questionnaire-based cross-sectional study was conducted among consenting Pharmacists at Ahmadu Bello University Teaching Hospital (ABUTH) and University of Nigeria Teaching Hospital (UNTH). Completed questionnaires were collated and analyzed using SPSS Version-25 with appropriate descriptive and inferential statistics. *P*-values less than 0.05 were considered to be statistically significant.

**Results:**

A total of 77 Pharmacists participated in the study, with 54(70.13%) being from ABUTH. In ABUTH, 15(33.3%) Pharmacists identified as being females, against 16(69.6%) in UNTH. Majority (40,95.2%) of the Pharmacists in ABUTH had less than 10 years working experience as against 8(34.8%) in UNTH. Forty-eight (88.9%) respondents knew the correct meaning of PMTCT. The Pharmacists in ABUTH and UNTH had mean knowledge scores of 58.70±2.88% and 52.17±6.19%, respectively; *t*(75)=1.094, *p*=0.760. In ABUTH and UNTH, 16(69.6%) and 22(42.3%) Pharmacists, respectively, strongly agreed that PMTCT can prevent future infections in the infants. Their mean attitude scores were 69.65±1.22% (ABUTH) and 74.09±1.68% (UNTH); *t*(73)=-2.063, *p*=0.487. For practice, 4(5.33%) Pharmacists in both hospitals very often dispensed PMTCT drugs, while 37(70.83%) counseled PMTCT treatment-naïve patients.

**Conclusion:**

The Pharmacists assessed in both hospitals had a fair knowledge of PMTCT services. Their attitudes to PMTCT was very good, although only a few of them had experience in providing care for PMTCT patients.

## Introduction

The highest mode of transmission of HIV infection in Africa is through heterosexual relationships (Pokharel *et al.*, 2012). In any such sexual relationships, the risk of transmission of HIV infection from males to females is higher than the reverse (Arulogun *et al.*, 2007) by about a 24-fold chance of higher transmission (Pokharel *et al.*, 2012). This results in a situation in which the incidence of HIV infection has been growing at a rate that is higher in women than men, especially among residents of sub-Saharan Africa where women make up to about 60 % of existing adult HIV infections ( The Joint United Nations Programme on HIV and AIDS, 2004; Anoje et al., 2012). It is estimated that approximately 5000 women are newly infected with HIV daily, out of which more than 3,000 die from AIDS-related illnesses (The Joint United Nations Programme on HIV and AIDS, 2002). Children who are born by HIV-infected women are thus at a high risk of acquiring the infection. It is estimated that almost all the HIV-infected children who are aged 15 years or less (90 % of about half a million annual cases) got the infection through mother-to-child transmission (Arulogun *et al.*, 2007). Without any form of intervention, there is a risk of an infant acquiring the virus from an infected mother that ranges from 15 % to 45 % (De Cock *et al.*, 2000; Hussein *et al.*, 2011; Pokharel *et al.*, 2012). The industrialized nations have a relatively lower risk (15 – 25 %) compared to the low and middle-income countries (LMIC) which has 25 % to 45 % probability of infection (Abdool Karim *et al.*, 2002), such that more than 40 % of all live births of children in communities in the LMIC are HIV infected in some of the countries (Pokharel *et al.*, 2012). Therefore, the epidemiology of HIV in children is directly related to that of women.

Prevention of mother-to-child transmission (PMTCT) is a HIV prevention strategy that is provided to HIV-positive women so that they could give birth to HIV-free children. In the strategy, the risk of mother-to-child transmission (MTCT) of HIV is reduced through well-designed programmes (Centers for Disease Control and Prevention, 2016) that involve the use of drugs and non-drugs related services. The drugs-related services involve the provision of treatment, care, and support to pregnant women who are infected with HIV, their children, as well as their families (Federal Ministry of Health, 2014; Centers for Disease Control and Prevention, 2016). it primarily has to do with the provision of antiretroviral drugs (ARVs) to the mothers at any point of pregnancy, delivery and breastfeeding, and a short course of ARVs for their babies. With the optimization of PMTCT, the risk of transmission of HIV from an infected mother to her child is reduced from 15 – 45 % to below 5 %.

The knowledge, attitude and practice (KAP) of healthcare professionals about PMTCT have been reported in literatures. A study conducted in Oyo State in Nigeria showed poor knowledge of PMTCT among healthcare workers. Only 37.5 % were shown to have a good level of knowledge of PMTCT (Aishat & Olubunmi, 2016). The findings of the study are not different from that of a similar study from Cameroon. The study showed poor knowledge, attitude, and practice of PMTCT among healthcare workers (Nkwabong *et al.*, 2018).. However, findings from South Africa showed a different result. A study conducted among nurses and doctors in South Africa revealed good knowledge and practice of PMTCT (Ogbonna *et al.*, 2016). Most of the respondents in the study reported good knowledge and practice. Respondents, however, demonstrated poor knowledge of the drug combinations and doses used in PMTCT. Another study from Nigeria also showed good knowledge, attitude and practice of PMTCT among Nurses/midwives (Ndikom & Onibokun, 2007)

To the best of the current researchers’ literature search, none of the studies mentioned or similar ones have specifically assessed the knowledge, attitude and practice of Pharmacists about PMTCT. This is despite the fact that they are in charge of the drugs that are dispensed to the patients in the pharmacological aspects of PMTCT interventions.

The aim of this study was to evaluate the knowledge, attitude and practice of PMTCT by Pharmacists in two hospitals in Nigeria.

## Materials and Methods

### Study Design

This survey adopted a cross-sectional design in the determination of the Pharmacists’ KAP of PMTCT.

#### Study Settings

The study was conducted among Pharmacists at Ahmadu Bello University Teaching Hospital (ABUTH), Zaria, Kaduna State, and the University of Nigeria Teaching Hospital (UNTH), Enugu, Enugu State. The two hospitals were conveniently selected so that the study would cover one hospital each in the northern and southern regions of Nigeria.

They were also the first hospitals in their zones to start HIV treatment and care, thus having the largest number of patients. ABUTH had 86 Pharmacists in its employ, while UNTH had 78 Pharmacists, at the time that the study was conducted.

### Study Sample

Pharmacists who were eligible from the two hospitals were recruited to participate in the study. To be eligible for the study, the respondent should have met the following criteria:

- Being a fully registered Pharmacist
- Having spent at least one year in the service of the hospitals.
- Having served at the HIV Clinic of the hospital for, at least, a month

In ABUTH, the number of Pharmacists that met the eligibility criteria was 55, while 25 Pharmacists in UNTH were eligible to participate in the study. Raosoft^®^ Online Sample Size Calculator was used to determine the sample size of the study from the two study sites. The margin of error in both populations was set at 5 %, while confidence interval level of 95 % was used. As per the default of the calculator, response distribution was set at 50 % for both populations. With the above data, a sample size of 49 was obtained for ABUTH, while 24 was obtained for UNTH. To make up for possible errors from the respondents, 10 % was added to each sample, such that the study sample size in ABUTH was 54 while all the eligible Pharmacists in UNTH (25) were used.

### Patients and Public Involvement

Patients and public were not involved in this study. Only the licensed Pharmacists that were practicing at the study sites were selected to participate in the study. Oral consent was obtained from the participants before they were provided the study instrument.

### Study Instrument

The questionnaire that was validated and used for the study had four sections: respondents’ sociodemographic characteristics, knowledge, attitude and practice of PMTCT. The questionnaire had acceptable face, content and construct validities, as well as good reliability.

### Study Procedure

All the Pharmacists in the hospital who met the eligibility criteria were approached to participate in the study. Upon the receipt of their oral consent, they were administered the questionnaire and politely requested to complete it on the spot and return. To assure confidentiality of the responses, the Pharmacists were made to return the completed questionnaires into a brown envelope and all were taken at the same time. A pen was provided for each Pharmacist that responded to the questionnaires as an incentive.

### Data Management and Analysis

The collated questionnaires were checked for completeness, by ensuring that the sociodemographic characteristics section was filled. They were then numbered serially and coded into Microsoft Excel (2016). before being exported into IBM SPSS Version-25 for data analysis.

Measures of central tendency (frequencies, percentages, means, etc.) and measures of dispersion (standard deviation, standard errors of mean (SEM), etc.) were used to describe the data. All responses were first presented as frequencies and percentages. Then the knowledge and attitude domains responses were scored for correctness and presented as means ± SEM. A group mean was also computed for each population, and it was used to divide the respondents into two groups of good knowledge and poor knowledge, and good attitude and poor attitude. T-test was used to compare the knowledge scores of the respondents of the two hospitals. The same comparison was done for the attitude scores. For all analyses, confidence interval was set at 95 %, with *p* values less than 0.05 considered statistically significant.

### Ethics Statement

The health research and ethics committees of ABUTH (Reference Number: ABUTHZ/HREC/S2/2017) and UNTH (Reference Number: UNTH/CSA/329/OL.5) granted ethical approval for this study to be conducted in their facilities.

## Results

### Study Instrument

The 27-item knowledge section of the questionnaire had a Kuder-Richardson Formular-20 value of 0.825. The 12-item attitude and 20-item practice sections of the questionnaire had Cronbach’s alpha values of 0.772 and 0.887 respectively.

### Sociodemographic Characteristics

Fifty-four Pharmacists completed the questionnaires in ABUTH. In UNTH, 23 Pharmacists returned the completed questionnaires. Pharmacists that were aged 26 - 35 years in ABUTH were 32 (60.4 %) while those that were aged 36 – 45 years in UNTH were 11 (47.8 %). B.Pharm/PharmD alone was the qualification of 47 (87 %) Pharmacists in ABUTH while 10 (43.5 %) had the same qualification in UNTH. In Table 1, the detailed socio-demographic characteristics of the Pharmacists are presented.

**Table 1:** Socio-demographic Characteristics of Pharmacists in ABUTH and UNTH

Characteristics		ABUTH		UNTH	

		Frequency	Percentage	Frequency	Percentage
Age (Years)	≤ 25	16	30.2	0	0.0
	26 - 35	32	60.4	2	8.7
	36 - 45	3	5.7	11	47.8
	46 - 55	1	1.9	8	34.8
	56 - 65	1	1.9	2	8.7
Gender	Male	30	66.7	7	30.4
	Female	15	33.3	16	69.6
Marital Status	Single	32	65.3	0	0.0
	Married	17	34.7	23	100
Religion	Christianity	10	19.6	23	100
	Islam	40	78.4	0	0.0
	Others	1	2.0	0	0.0
Highest Qualification	B.Pharm and FPCPharm	0	0.0	7	30.4
	B.Pharm/PharmD	47	87	10	43.5
	M.Sc/MPharm	5	9.3	3	13.0
	PhD and FPCPharm	1	1.9	1	4.3
	MPharm and FPCPharm	1	1.9	1	4.3
	Ph.D	0	0	1	4.3
Years of experience	≤ 10	40	95.2	8	34.8
	10 - 20	1	2.4	14	60.9
	≥ 20	1	2.4	1	4.3
Attending to HIV/AIDS Patients	Never	12	24.0	5	21.7
	Rarely	17	34.0	10	43.5
	Sometimes	11	22.0	4	17.4
	Often	7	14.0	0	0.0
	Very Often	2	4.0	2	8.7
	Cannot Remember	1	2.0	1	4.3
Training on HIV/AIDS after Graduation	None	36	87.8		
	HAART	2	4.9	13	56.5
	PMTCT	1	2.4	3	13
	TB and HIV	0	0.0	4	17.3
	Counseling	2	4.9	3	13
Satisfaction with Income	Not Sufficient	13	26.0	14	60.9
	Meets Needs	29	58.0	8	34.8
	Allows Saving	8	16.0	1	4.3
Overall Satisfaction with Job	Not at all Satisfied	4	7.8	2	8.7
	Slightly Satisfied	5	9.8	7	30.4
	Moderately Satisfied	31	60.8	8	34.8
	Very Satisfied	11	21.6	5	21.7

### Knowledge of PMTCT

The Pharmacists in ABUTH had a knowledge of PMTCT score of 58.70 ± 2.88 % while their UNTH counterparts had a score of 52.17 ± 6.19 %.: t (75) = 1.094, *p* = 0.760 (2-tailed). The question with the most correct response from Pharmacists in ABUTH was on the meaning of PMTCT with a mean score of 0.89 + 0.04:48 (88.90 %) Pharmacists got the right response. For Pharmacists in UNTH, the most correct response was about the commencement of PMTCT at the second trimester (0.74 ± 0.09): 17 (73.2 %) Pharmacists knew the right reponse the question. The responses to all the questions are presented in Table 2. In Table 3, the groupings of the Pharmacists into poor and good knowledge using their respective overall mean scores are presented.

**Table 2:** Pharmacists’ Knowledge of PMTCT in both Hospitals

Questions		ABUTH				UNTH			

		n	%	Mean	SEM	n	%	Mean	SEM
PMTCT at second trimester	Incorrect	20	37.0	0.63	0.066	6	26.1	0.74	0.094
PMTCT at fourth trimester	Incorrect	28	51.9	0.48	0.069	9	39.1	0.61	0.104
PMTCT at child delivery	Incorrect	30	55.6	0.44	0.068	17	73.9	0.26	0.094
PMTCT at postnatal period	Incorrect	24	44.4	0.56	0.068	9	39.1	0.61	0.104
ARVs in early pregnancy	Incorrect	11	20.4	0.80	0.055	3	13.0	0.87	0.072
PMTCT while breastfeeding	Incorrect	25	46.3	0.54	0.068	8	34.8	0.65	0.102
Pregnancy as HAART indication	Incorrect	23	42.6	0.57	0.068	5	21.7	0.78	0.088
Breastfeeding implies no PMTCT	Incorrect	21	38.9	0.61	0.067	11	47.8	0.52	0.106
Current 1st line PMTCT drugs for mothers	Incorrect	40	74.1	0.26	0.060	19	82.6	0.17	0.081
PMTCT ARV for Child	Incorrect	19	35.2	0.65	0.066	12	52.2	0.48	0.106
Dose for < 6 weeks old and < 2.5Kg	Incorrect	29	53.7	0.46	0.068	18	78.2	0.22	0.088
Dose for < 6 weeks old and ≥ 2.5Kg	Incorrect	29	53.7	0.46	0.068	18	78.3	0.17	0.081
Dose for > 6 weeks to 6 months	Incorrect	50	92.6	0.07	0.036	14	60.9	0.39	0.104
Dose for > 6 months to 9 months	Incorrect	37	68.5	0.31	0.064	20	86.9	0.13	0.072
Dose for > 9 months to 12 months	Incorrect	36	69.1	0.35	0.066	22	95.7	0.00	0.000
Bases of ARV dosing in child	Incorrect	19	35.2	0.65	0.066	9	39.1	0.61	0.104
Period of ARV administration in child	Incorrect	48	88.9	0.11	0.043	20	87.0	0.13	0.072
Mother not on HAART a special situation	Incorrect	17	31.5	0.69	0.064	8	34.8	0.65	0.102
Starting HAART at breastfeeding a special situation	Incorrect	19	35.2	0.65	0.066	13	56.5	0.43	0.106
Co-trimoxazole contraindication in exposed child	Incorrect	23	42.6	0.57	0.068	9	39.1	0.61	0.104
Stopping of HAART by the prescriber	Incorrect	54	100.0	0.00	0.000	23	100.0	0.00	0.000

**Table 3:** Grouping of Pharmacists into Knowledge Categories and the Sources of their Knowledge

Characteristics		ABUTH		UNTH	

		n	%	n	%
Category of Knowledge	Poor Knowledge	25	48.1	5	21.7
	Good Knowledge	27	51.9	18	78.3
Source of Knowledge	National Guideline	5	9.3	0	0.0
	Training/Workshop	13	24.1	4	17.4
	Colleagues	9	16.7	2	8.7
	Mass Media	2	3.7	1	4.3
	Others	3	5.6	0	0.0
	National Guideline + Training	1	1.9	1	4.3
	Guideline + Training + Colleagues	1	1.9	0	0.0
	Guideline + Colleague	1	1.9	0	0.0
	Training + Colleagues	0	0.0	1	4.3
	Colleague + Mass Media	0	0.0	1	4.3

### Attitude towards PMTCT

The overall mean score of the Pharmacists in UNTH was 74.09 ± 1.68 %, while the Pharmacists in ABUTH had an overall mean attitude score of 69.65 ± 1.22 %: t (73) = -2.063, *p* = 0.487 (2-tailed). Of the 23 Pharmacists in ABUTH, 16 (69.6 %) *strongly agreed* that they believed that PMTCT could prevent future HIV infections in the infants, while 22 (42.3 %) Pharmacists from UNTH had a similar response for the same question. The responses of the Pharmacists from the two hospitals are presented in Table 4.

**Table 4:** Pharmacists’ Attitude about PMTCT in both Hospitals

Questions	Hospital	SD	D	N	A	SA	Mean ± SEM

		Frequency (Percentage)					
Prevents against future HIV infection in the child	ABUTH	8 (15.4)	5 (9.6)	3 (5.8)	14 (26.9)	22 (42.3)	3.71 ± 0.206
	UNTH	1 (4.3)	0 (0.0)	0 (0.0	6 (26.1)	16 (69.6)	4.57 ± 0.187
Continue prophylactic therapy even when not breastfeeding	ABUTH	4 (7.4)	9 (16.7)	7 (13.0)	25 (46.3)	9 (16.7)	3.48 ± 0.160
	UNTH	2 (8.7)	3 (13.0)	2 (8.7)	8 (34.8)	8 (34.8)	3.74 ± 0.276
Prevent horizontal transmission of HIV	ABUTH	11 (20.4)	21 (38.9)	13 (24.1)	6 (11.1)	3 (5.6)	2.43 ± 0.151
	UNTH	9 (39.1)	6 (26.1)	3 (13.0)	2 (8.7)	3 (13.0)	2.30 ± 0.298
No drug therapy problem stops PMTCT	ABUTH	4 (7.4)	12 (22.2)	11 (20.4)	17 (31.5)	10 (18.5)	3.31 ± 0.160
	UNTH	0 (0.0)	5 (21.7)	4 (17.4)	7 (30.4)	7 (30.4)	3.70 ± 0.239
Adherence is important	ABUTH	32 (59.3)	16 (29.6)	3 (5.6)	2 (3.7)	1 (1.9)	1.59 ± 0.123
	UNTH	17 (73.9)	4 (17.4)	2 (8.7)	0 (0.0)	0 (0.0)	1.35 ± 0.135
Stay far away from a PMTCT patient while dispensing drugs to her	ABUTH	2 (3.7)	4 (7.4)	7 (13.0)	9 (16.7)	32 (59.3)	4.20 ± 0.157
	UNTH	1 (4.3)	1 (4.3)	0 (0.0)	1 (4.3)	20 (87.0)	4.65 ± 0.214
Much counseling not necessary	ABUTH	0 (0.0)	5 (9.3)	4 (7.4)	10 (18.5)	35 (64.8)	4.39 ± 0.133
	UNTH	0 (0.0)	1 (4.3)	2 (8.7)	5 (21.7)	15 (65.2)	4.48 ± 0.176
Expired drugs can be dispensed in PMTCT	ABUTH	1 (1.9)	9 (16.7)	17 (31.5)	15 (27.8)	12 (22.2)	3.52 ± 0.147
	UNTH	0 (0.0)	3 (13.0)	3 (13.0)	9 (39.1)	8 (34.8)	3.96 ± 0.213
All drugs counseling by nurses	ABUTH	1 (1.9)	4 (7.4)	7 (13.0)	13 (24.1)	29 (53.7)	4.20 ± 0.143
	UNTH	0 (0.0)	1 (4.3)	0 (0.0)	7 (30.4)	15 (65.2)	4.57 ± 0.157
PMTCT not an emergency in infants	ABUTH	1 (1.9)	10 (18.5)	10 (18.5)	16 (29.6)	17 (31.5)	3.70 ± 0.158
	UNTH	0 (0.0)	5 (21.7)	4 (17.4)	6 (26.1)	8 (34.8)	3.74 ± 0.245

SD: Strongly Disagree, D: Disagree; N: Neutral; A: Agree; SA: Strongly Agree.

*Practice of PMTCT*

For practice, 4 (5.33 %) Pharmacists in both hospitals *very often* dispensed PMTCT drugs, while 37 (70.83 %) counseled PMTCT treatment-naïve patients. In ABUTH, 3 (5.9 %) Pharmacists *very often* dispensed PMTCT drugs while 1 (4.3%) Pharmacist did same in UNTH. Also, in ABUTH, 47 (95.9 %) Pharmacists had *never* changed a patient’s drugs without consulting the prescribers while 22 (95.7 %) Pharmacists in UNTH had *never* changed a patient’s prescription. Whereas 37 (68.5%) Pharmacists reported that they counselled treatment-naïve PMTCT patients, 14 (60.9%) in UNTH gave the same response. For treatment-experienced patients, 37(75.5%) Pharmacists in ABUTH counselled them on their medications while 14 (60.8%) did same in UNTH.

The responses for practice questions in the two hospitals are presented in Table 5.

**Table 5:** Pharmacists’ Practice of PMTCT in both Hospitals

Practice	Hospital	Never	Rarely	Sometimes	Often	Very Often	Can’t Remember

Frequency (Percentage)							
How often do you dispense drugs for PMTCT patients?	ABUTH	16(31.4)	16(31.4)	8(15.7)	4(7.8)	3(5.9)	4(7.8)
	UNTH	12(52.2)	5(21.7)	2(8.7)	1(4.3)	1(4.3)	2(8.7)
Frequency of intervening with adverse drugs effects	ABUTH	21(41.2)	13(25.5)	11(21.6)	1(2.0)	2(3.9)	3(5.9)
	UNTH	9(39.1)	6(26.1)	4(17.4)	1(4.3)	-	3(13.0)
Frequency of intervening with drug interaction	ABUTH	22(43.1)	10(19.6)	8(15.7)	6(11.8)	2(3.9)	3(5.9)
	UNTH	10(43.5)	4(17.4)	4(17.4)	1(4.3)	1(4.3)	3(13.0)
Frequency of intervening with drugs for comorbidities	ABUTH	20(39.2)	9(17.6)	9(17.6)	7(13.7)	2(3.9)	4(7.8)
	UNTH	9(39.1)	6(26.1)	4(17.4)	1(4.3)	0(0.0)	3(13.0)
Frequency of intervening with adherence issues	ABUTH	20(40.0)	9(18.0)	5(10.0)	10(20.0)	4(8.0)	2(4.0)
	UNTH	8(34.8)	2(8.7)	6(26.1)	3(13.0)	1(4.3)	3(13.0)
Frequency of intervening with drugs not indicated	ABUTH	27(52.9)	8(15.7)	6(11.8)	3(5.9)	2(3.9)	5(9.8)
	UNTH	10(43.5)	6(26.1)	3(13.0)	1(4.3)	0(0.0)	3(13.0)
Frequency of intervening with dosage adjustment	ABUTH	25(50.0)	13(26.0)	4(8.0)	3(6.0)	2(4.0)	3(6.0)
	UNTH	10(43.5)	5(21.7)	2(8.7)	3(13.0)	0(0.0)	3(13.0)
Frequency of intervening with other reasons	ABUTH	14(73.7)	1(5.3)	3(15.8)	0(0.0)	0(0.0)	1(5.3)
	UNTH	7(30.4)	0(0.0)	0(0.0)	0(0.0)	3(13.0)	13(56.5)

## Discussion

### Key Findings and Interpretation

The response rate of the Pharmacists to the study was impressive from the two hospitals: ABUTH and UNTH. The majority of the Pharmacists in both hospitals were of middle age, while a vast majority only had first-degree pharmacy qualification. The Pharmacists in both hospitals had a good knowledge of PMTCT. However, the Pharmacists in ABUTH had a slightly higher knowledge score than their counterparts in UNTH, although the difference was not statistically significant. They also scored highly in most questions in the knowledge section. For the attitude section, the Pharmacists’ performance was outstanding, although those in UNTH had a higher but not statistically significant score compared to their ABUTH counterparts. On the practice of PMTCT, not many Pharmacists *very often* dispensed PMTCT drugs to patients in the two hospitals.

### Comparison and Implications

The socio-demographics findings of the present study are comparable to those of a cross-sectional study conducted among Pharmacists in three government hospitals in Kedah Malaysia. The authors used a self-administered 43-item questionnaire and found out that the majority of the respondents were single, females, and aged between 26-30 years (Khan & Baig, 2013). These socio-demographic data are similar to those of majority of the respondents from UNTH. However, the majority of the respondents in ABUTH were single, unlike those in UNTH who were mostly married.

Other studies on KAP of PMTCT are about healthcare professionals other than Pharmacists, hence limiting comparison of the findings of this study. In most of the studies, PMTCT services were rendered by more married females and thus agree with Okoli and Lansdown, who posit that there is low involvement of males in PMTCT services. This is probably because the concept of PMTCT has been considered to be a woman-child affair and does not seem to accommodate any form of male participation (Okoli & Lansdown, 2014). The findings also agree with Thomson *et al*. who argue that males were a major influence in the risk assessment of PMTCT, but were generally unaware of PMTCT services (Thomson *et al.*, 2018). Therefore, the successful scale-up of PMTCT services needs to include more male healthcare providers in a study carried out at Burkina-Faso to review the achievements and challenges in the PMTCT of HIV, it was revealed that an acceleration in the uptake of PMTCT services in Burkina- Faso depended upon the inclusion of males. It also stated that the integration of men as partners in PMTCT services was critical to the success of PMTCT in Burkina-Faso (Linguissi *et al.*, 2019).

Other factors that have been mentioned as barriers to PMTCT services delivery are religious beliefs, family disruption, and gender. Thus, successful implementation of PMTCT policies must eradicate these challenges and create an environment for the collective contribution of all sexes to PMTCT services.

The majority of the respondents from both institutions had correct knowledge on the full meaning of PMTCT and the caution in the use of ARVs in early pregnancy. They demonstrated very fair and, sometimes, poor knowledge in many other essential concepts of PMTCT. The Pharmacists who responded in the Malaysian study revealed that the majority of the pharmacists were found to be well aware of the causes of HIV/AIDS, but had very erroneous knowledge of its transmission routes (including MTCT), prevention strategies (including PMTCT), and methods of social association with persons living with HIV (Khan & Baig, 2013). To enhance the knowledge and optimize the successful implementation of PMTCT services in Nigeria, there is the need to take several interventional steps, including continuous education and training

Since primary care Pharmacists see their patients between 1.5 and 10 times more frequently than the patients see their primary care Physicians (Tsuyuki *et al.*, 2018), they are crucial to the effective integration of the provision of primary ante-natal services to HIV-positive mothers in pharmacy services. Moreover, the accessibility of the Pharmacists provides a unique opportunity to deliver optimal medication utilization for PMTCT patients (Blouin, 2017).

The positive attitude towards PMTCT among Pharmacists in UNTH compared to their ABUTH counterparts is probably as a result of the greater number of training that the former had received on the concept of PMTCT and other areas of HIV, as documented in the results. This is plausible as studies have long identified a correlation between the experience of training and the attitude of the trainee (Yan & Ming, 1992). Thus, efforts to increase the training of Pharmacists on emerging concepts of PMTCT and other areas of HIV would be invaluable in enhancing their attitude and PMTCT service delivery. Some of the negative attitudes demonstrated by Pharmacists from both institutions in this study correlate with some researches on the attitude of Pharmacists to PMTCT. The majority of the respondents of the Malaysian survey had negative attitudes, and a significant population of the respondents even held extremely negative attitudes. The cross-sectional study reported that many respondents endorsed social isolation as a measure to prevent HIV/AIDS. This study, therefore, agrees with Wanyenze *et al.*, who suggested that more attention is needed to enhance provider-client communication because communication with PMTCT providers was associated with Antenatal Care uptake of PMTCT services among people living with HIV (PLHIV) in care (Wanyenze *et al.*, 2018). There is the possibility that the negative attitudes of respondents in this study may be attributed to their dissatisfaction with their income as the majority of the respondents at UNTH noted that their income did not meet their needs. The impact of human resources and the effect of a low-morale workforce on the provision of adequate PMTCT services have been mentioned in a study (Okoli & Lansdown, 2014).

On the practice of PMTCT by the Pharmacists, respondents from both institutions were neutral on interventions in drug therapy problems while dispensing PMTCT ARTs such as adverse drug effects and dosage adjustment problems. Since Pharmacists do not have the legal backing to change a prescription without recourse to the prescribers, it was wrong for some respondents to have ever done so. Since the majority of respondents only rarely and sometimes never dispensed drugs for PMTCT patients in both hospitals, there is the need to involve more Pharmacists in their HIV clinics.

## Conclusion

The Pharmacists assessed in both hospitals had a fair knowledge of PMTCT, with majority knowing the meaning of PMTCT and its components. Their attitude to PMTCT was very good, but they had a poor attitude to a few aspects of PMTCT care. Only a few of the Pharmacists had experience in providing care for PMTCT patients.

### Conflict of Interest

The authors declare that they have no conflict of interest related to this study.

**Abbreviations:**
ABUTH: Ahmadu Bello University Teaching Hospital;
AIDS: acquired immune deficiency syndrome;
ARVs: antiretroviral agents;
HIV: human immunodeficiency virus;
IBM SPSS: International Business Machine Statistical Products and Services Solution;
LMIC: low and middle-income countries;
MTCT: mother-to-child transmission;
PLHIV: people living with HIV;
PMTCT: prevention of mother-to-child transmission;
SEM: standard errors of mean;
UNTH: University of Nigeria Teaching Hospital.
